# Using system dynamics for collaborative design: a case study

**DOI:** 10.1186/1472-6963-7-123

**Published:** 2007-08-02

**Authors:** Marie Elf, Mariya Putilova, Lena von Koch, Kerstin Öhrn

**Affiliations:** 1 Chalmers University of Technology, Göteborg, Sweden; 2 Centre for Clinical Research Dalarna, Falun, Sweden; 3 IT University, Göteborgs Universitet , Göteborg, Sweden; 4 Karolinska Institutet, Department of Clinical Neurosciences, Stockholm, Sweden; 5 Dalarna University Department of Health and Social Sciences, Falun, Sweden

## Abstract

**Background:**

In order to facilitate the collaborative design, system dynamics (SD) with a group modelling approach was used in the early stages of planning a new stroke unit. During six workshops a SD model was created in a multiprofessional group.

**Aim:**

To explore to which extent and how the use of system dynamics contributed to the collaborative design process.

**Method:**

A case study was conducted using several data sources.

**Results:**

SD supported a collaborative design, by facilitating an explicit description of stroke care process, a dialogue and a joint understanding. The construction of the model obliged the group to conceptualise the stroke care and experimentation with the model gave the opportunity to reflect on care.

**Conclusion:**

SD facilitated the collaborative design process and should be integrated in the early stages of the design process as a quality improvement tool.

## Text

Designing spaces for patients is an important quality improvement process in which various participants are involved, such as health care professionals, building planners, health care managers and architects. Evidence shows that the physical space has a major impact on the care processes and the patient's health [1,2]. In order to create a patient-centred care process the focus needs to be on communication, collaboration and shared decision-making with the patient [3-5]. Subsequently, quality improvements in health care are achieved by designing physical spaces that support patient-centred care by offering specific spaces dedicated to communication and meetings between professionals, patients and relatives [2,6,7]. The participants in the design process thus need to consider not only the construction of a physical space but also the whole context of care if an optimal design is to be achieved [6,8,9]. The initial stage of the design process should focus on the goals for care, health care processes and the activities that are to take place in the envisaged building.

Designing is an iterative problem-solving process in which the object gradually emerges through a procedure of identifying problems, structuring knowledge and generating and testing design solutions [10-12]. The early stage of the process has been regarded as being specifically important, as it is the stage in which the goal of the design should be expressed [13]. Studies have shown that communication deficit at an early stage was caused by the different values, concepts and priorities of the participants involved [14-16]. Flaws in the first stage in the process may result in poor quality of the space and failure to meet the users' expectations. It may even lead to the need for redesigning and rebuilding with additional costs [15,16].

Recently, a collaborative design process has been suggested as an effective design method in which issues about how space and work processes exert a mutual influence can be exposed [16-18]. Collaborative design entails active communication and collaboration between key participants on order to establish design goals. The approach focuses on reflection, dialogue and interaction between key participants as a way of learning and understanding the context [16-19]. An important aspect is to work with conceptual models to create a common understanding between construction and various health care professionals how the system works [16].

### System dynamics

**Figure 1:**
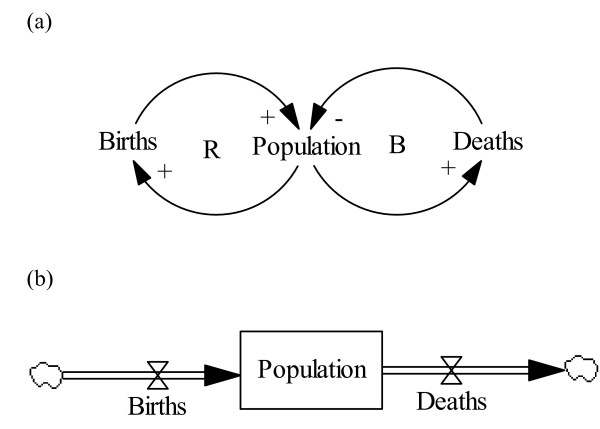
(a) Causal loops diagram (b) Stock and flow diagram.

System dynamics (SD) is used to build models of a real world and to study how its (real world's) structure produces dynamic behaviour over time [20,21]. The method allows to experiment with changes in a model, which is impossible to perform in the real world. The method combines both qualitative and quantitative aspects to explore, realise and communicate complex ideas [20,21]. The qualitative part entails the creation of causal loop diagrams (CLD), as depicted in figure 1(a), in which variables are mapped in a cause and effect relationship pattern, which creates the hypothesised dynamic structure of the system.

A cause and effect relationship can either change the behaviour in the same direction (indicated using a plus sign) or in the opposite direction (indicated using a minus sign). A loop with a B indicates that the loop is balanced and seeking stability in the system, and with a R that the loop is reinforcing [22].

The quantitative aspect involves the development of a computer model based upon a "stock and flow diagram" (SFD) as illustrated in figure 1(b), and equations which depict interrelated variables in the system. Stock variables (rectangles) represents the state variables and are the accumulations in the system. Flow variables (valves) alter the stocks by filling or draining the stocks. Arrows point the causal relation between two variables and also reflect the flow of information within the model structure [21].

Such models can be used for "what-if" questions to experiment with alternative scenarios by changing the values of the variables in the model which, when run, produce output (a simulated dynamic behaviour). The output of the simulation can then be compared with the real world [23].

The level of detail in the model is related to the project questions, which create the boundaries of the model and determine which variables should be included. Furthermore, all variables are defined explicitly, which makes the procedure transparent since all variables and their functions are visible.

Previous works using models for design process have often been set on an administrative level and have mainly adopted an industrial view of processes (traditionally well defined and easy to control and predict). The focus of these studies has been on queue situations and bottlenecks within the system [24-30]. This is also typical for many of the SD models previously created for health care in which SD has been used to analyse patient pathway, information flow and resources used [31-33]. The method has also been used to analyse feedback consequences of waiting times [34,35]. However, the clinical care process is complex and includes many qualitative variables. Few studies have used SD in order to identify and analyse factors that drive quality in health care [36,37].

When a new clinic for stroke care was planned at a Swedish hospital a collaborative approach using SD with a group modeling approach was employed at an early stage in the design process. The aim was to facilitate the discussions about the stroke care process as a base for decisions about the physical design.

## Aim

The aim of the study was to explore to what extent and how the use of SD contributes to the collaborative design process in the early stages of the design of a new health care environment in which various stakeholders are involved.

## Methods and materials

### Study design

**Figure 2:**
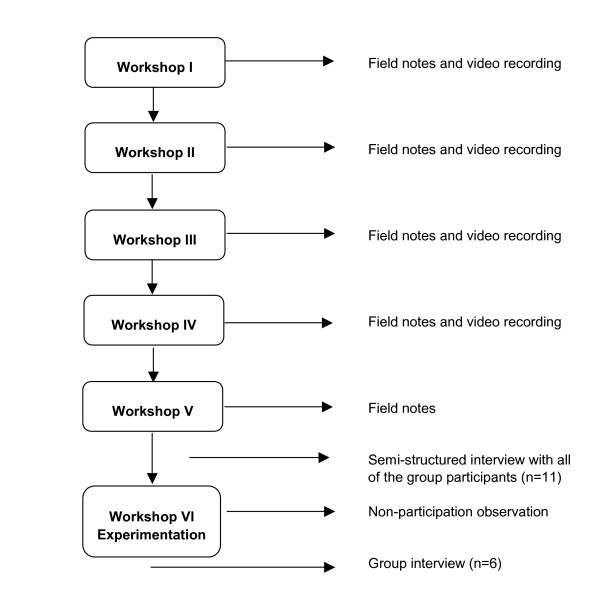
The research process.

This was a case study [38,39] using qualitative and quantitative data. The case unit analysed was the collaborative design process using SD with a group-modelling approach. The research process is outlined in figure 2. The data collection included:

• A video recording of the first 4 workshops (which were transcribed)

• Field notes written by the project leader after each workshop with a focus on what had occurred during the meetings and reflections on the activities and topics of discussion between the participants in the group.

• Structured interviews with each participant performed after the fifth workshop by the interviewer (a person not involved in the collaborative design process) filling in the questionnaire together with the participants on a five-point Likert scale, ranging from Totally disagree (1) to Totally agree (5) regarding a) the overall success of the meetings, b) insights into the care process c) communication and creation of a shared vision.

• Responses from the participants to open-ended questions regarding experiences of the modelling process written down by the interviewer.

• Non-participant observations by two independent observers who concurrently made field notes on the activities and communication between the participants in the group during the final workshop. The notes were transcribed.

• A semi-structured group interview was performed after the final workshop. The participants were asked to describe their experience of SD in the collaborative design process. Six of the participants took part in the interview, which lasted for approximately one hour and was audiotaped and transcribed.

### Setting and participants

The present project was integrated into a planning and construction process (design process) for a new stroke unit at a Swedish hospital. The design of the present stroke unit was old-fashioned with rooms for patients containing up to six beds. Long corridors connected the rooms. The staff and managers did not feel that the unit supported optimal stroke care.

The group consisted of ward managers (nurses, n = 2), a clinical stroke nurse, a physiotherapist, nursing aides (n = 2), building planners (n = 3, one of whom was also a nurse) and architects (n = 2). The project leader (nurse ME) and the modeller (MP) were also in the group. At the first meeting the head of the clinic (physician) was present to sanction the project and participate in the discussions.

### The modelling intervention

A group modelling approach was used and the SD model was created in collaboration with the group. The participants were involved at all stages of the modelling process except for the technical formulation of equations in the model. The project consisted of six workshops (each lasted two to three hours) and spanned a period of 18 months. The project leader and the SD modeller intervened and worked with the group directly by contributing with modelling knowledge. The group-modelling consisted of four stages 1) problem identification and model purpose, 2) conceptualisation by using CLD, 3) computer model formulation and parameterisation, 4) experimentation and reflections. The process was highly iterative.

#### Problem identification and model purpose

During the first workshop the group identified the overall challenges of the stroke care process and the problems at the existing unit. The purpose of the modelling project and the model boundaries were discussed and the group identified key variables to be considered for inclusion in the model. Donabedian's [40] quality model was used to sort out the variables provided by the group. The quality model includes: outcome quality (outcomes essential to achieve), process quality (activities necessary to perform in order to achieve the defined outcomes) and structural quality (the physical attributes necessary to achieve the goal, e.g. equipment, laws or staff).

#### Conceptualisation

The key variables considered for inclusion in the model were discussed and defined more in detail throughout the project based on scientific literature and experiences of the stroke care process.

#### Computer model formulation

At the second workshop CLD was introduced gradually, starting with two concepts and their relationship and thereby creating small parts of the CLD. Thereafter additional concepts were included in the model. Each connection between the variables was discussed thoroughly before decisions were made based on evidence from the literature. At the third workshop the creation of the mathematical model was introduced. Most of the parameterisation was performed between workshops by the project leader (ME) and the modeller (MP). The model (the CLD) is presented in Appendix 1.

#### Experimentation and reflections

**Figure 3:**
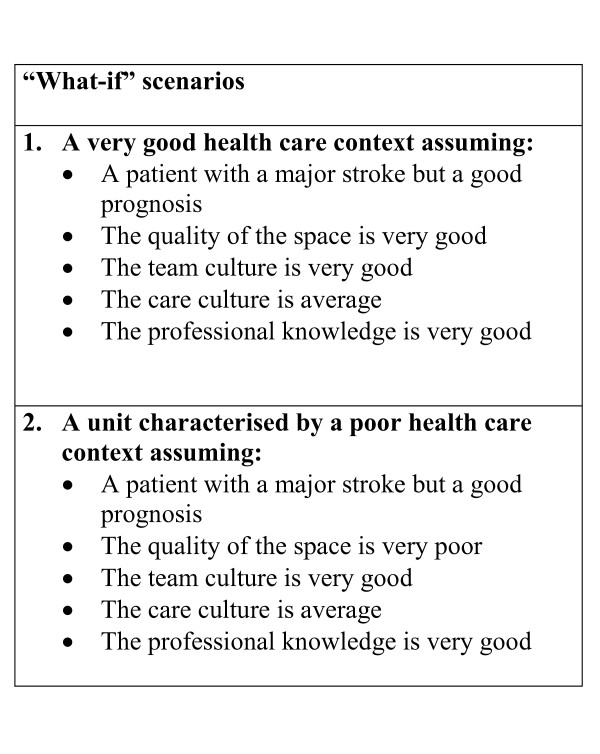
Examples of scenarios created at the experimentation workshop. The parameters in the model could be altered from 0 (very poor quality) - 1 (very good quality).

**Figure 4:**
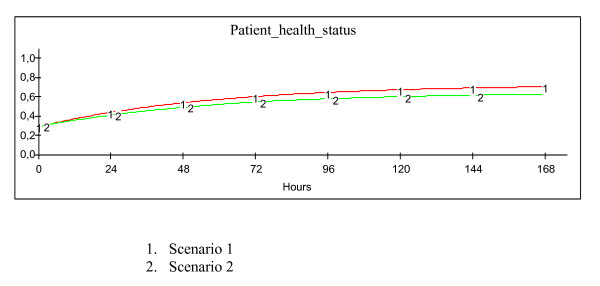
Outcomes from the scenarios.

"What if" analyses were performed iteratively during the modelling process although the last workshop was dedicated to experimentation. The group specified scenarios in collaboration with the project leader and the modeller, using explicit definitions and grading of the variables ranging from very poor to very good. Various space design policies were discussed and generated. Examples of two of the scenarios created at the last workshop are presented in Figure 3 and the outcomes of the scenarios are depicted in Figure 4.

### Data analysis

The trustworthiness of the findings was enhanced through triangulation. The structured interviews were analysed using descriptive statistics. The content of the video recordings and the group interviews, the field notes and the answers to the open-ended questions were analysed with regard to activities at the workshops, the behaviour of the participants and communication in the group as well as statements reflecting the usefulness of SD modelling in the collaborative design process. The first and the fourth authors analysed the content of the field notes, video recording and interviews separately. There was total agreement between the two persons who analysed the data. Two of the participants in the design group confirmed the findings.

### Ethical considerations

The head of the department in which the design process took place approved the study. The group participants announced their interest in participating in the project after an initial information meeting. Informed consent for videotaping, observations and interviews were obtained from the participants.

## Findings

The results showed that SD contributed to a collaborative design, primarily by facilitating an explicit description of patient-centred stroke care. The method also supported profound conceptualisation of stroke care by discussions of the key variables, which contributed to a dialogue and a joint understanding. Moreover, the opportunity to experiment with the model supported reflection on the significance of the different variables in the model and of their importance to the patient's health. Certain obstacles were encountered in the project, which hampered the group-modelling design process.

### Explicit description of stroke care from a patient perspective

Evidence from the field notes and videotapes showed that the model developed became an interactive tool in which the stroke care process was discussed between participants, explained and incorporated into the design process. At the first workshop the caring professionals expressed a need for a stroke unit with more beds ("more capacity, better care quality"). The dimension of the space was initially the core issue but, despite that, the building planners urged the caring professionals to express their demands beyond their requests regarding dimension. However, during the course of the project the issues of dimension declined in importance and quality issues in stroke care became more apparent. The discussions focused on the patient's needs and the variables that contribute to health.

"Yes, by using the model to show that we were on the right track regarding quality variables, for example that communication with the patient is one important variable in stroke care,.... the modelling project supported our previous discussions about stroke care."

During the first interview the participants (n = 11) stated that the modelling had supported an understanding of other participants' ideas and viewpoints (Table 1).

**Table 1:** Answers from the structured interview reported with median and the mode.

**Questions**	**md/mode**
In general, I think that the meetings have been successful	**4/5**
Modelling is an effective way to make a problem visible in an organisation	**4/4**
On the whole I support the conclusions and findings at the workshops	**5/5**
The modelling workshops supported an understanding of other people's viewpoints and ideas	**4/5**
Modelling provides a better opportunity to share visions between players compared with other meetings	**4/4**
As a result of the modelling workshops we have reached a shared vision of problems in our organisation	**3/3**
The participants' visions have come closer due to the modelling workshops	**4/4**
The causal diagram made communication between the participants comprehensible	**4/4**
Modelling produces better communication between the participants compared with other meetings	**4/4**
The causal diagram was developed from the participants' opinions and ideas	**5/5**
The modelling workshops have given me better insight into how the different parts in stroke care are related to each other	**3/4**

In the final interview (n = 6), the participants stated that the use of SD had contributed to an explicit description of stroke care and that the modelling process had made tacit knowledge more evident.

"I realised that tacit knowledge becomes explicit. The simulation project gives another dimension to the facts already known. Things we knew beforehand were confirmed."

"It feels like there is a lot of knowledge behind the model that has been developed, which makes it serious. It is not a model where you can push a button and obtain a figure."

### Conceptualisation of stroke care

**Figure 5:**
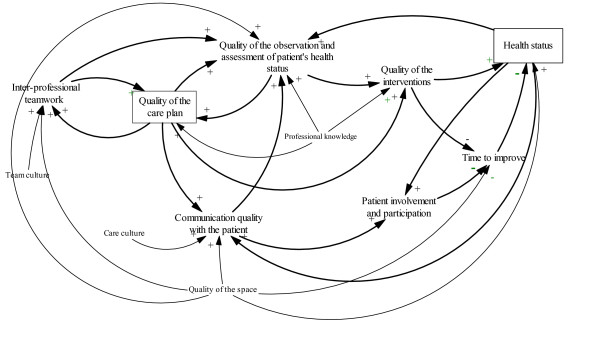
A CLD of stroke care.

The CLD assisted the conceptualisation of stroke care according to the structured interview, Table 1, the field notes and content of the workshops. The CLD allowed evidence-based variables in stroke care to be integrated into a transparent dynamic model (see figure 5). Several of the participants had evidence-based knowledge of stroke care, which they referred to during the process. In addition, the project leader brought additional scientific evidence to the group in response to issues raised during the workshops. By creating the CLD the group was forced to define all variables explicitly and to connect them in a comprehensible cause and effect model. At the end of the project, when the model was refined, only the most important variables remained.

From the field notes and the video recording it was revealed that throughout the project the participants frequently returned to the concepts within the model during the discussions.

"I believe that teamwork is more important and had a greater impact on the patient's health progress than observation and assessment. If you have efficient teamwork it contributes to communication in the team and the exchange of information between the professionals."

"The meaning of teamwork should be defined differently. We should talk about high quality teamwork and what that includes."

A major concern was the concept of health, which was discussed throughout the project.

"How can health be defined? Is it the length of stay in hospital? Is it subjective experiences of health or objective health?"

Based on the field notes and the videos it was obvious that the group could not accept the commonly used assessment of health as "treatment completed at the emergency clinic", which is more an assessment of the emergency clinic's obligation and responsibility for the individual patient. At the second workshop the project leader presented the WHO health model (International classification of functioning, disability and health) (ICF) [41], which formed the basis for further discussions in the group and later on for the definition of health in the refined model.

In the first interview many participants stated that they were sceptical about working with the CLD. They admitted that the diagram had been developed jointly in the group (Table 1) and many of them considered the CLD to be helpful in achieving comprehensible communication (Table 1). Despite this, there were several negative comments:

"Too many lines and arrows to be useful."

"The method is too technical to be useful."

"The diagram is difficult to understand and has to be explained to be useful."

However, evidence from the field notes and videotapes challenged this attitude and showed that throughout the project the CLD supported reflective behaviour in the group by supplying a framework for further discussions and analysis of the care process.

### Experimentation as support for reflections on care

From the field notes, the videos and the interviews it was revealed that the key to greater understanding was the opportunity to experiment with the model. The experimentation supported reflection on the significance of the different variables contained in the model and their importance to the patient's health. It was also noticeable that the participants discussed stroke care as a dynamic process.

From the field notes of the non-participant observations it was noticed that during experimentation the participants had no difficulty in formulating scenarios or interpreting the results. The scenarios generated lively discussions in the group, including questions of clarification about the model but also about the existing stroke care in their own clinical setting. The participants considered the results of the simulation to be logical but on certain occasions during the workshop they questioned the structure of the system and wanted to discuss the magnitudes of the relationships, such as:

"What does the relationship between health and communication look like in the model?"

"I believe that the relationships between the quality of the space and the patient's health are stronger than the model shows...the space has a greater impact on the possibility to make an optimal observation and assessment of a patient's needs."

The experimentation gave the group the opportunity to further analyse the structure of the model at the same time the simulations were performed. In addition, the simulation gave rise to discussions about the definitions of complex concepts such as health and care planning. When the group asked to define the scenarios, they were forced to reflect on their own care setting. At the beginning of the last workshop, they considered their teamwork to be excellent but during the simulation they modified their opinion when the definition of teamwork quality was explicit. Moreover, they reflected on the relationship between the space and its impact on the quality of the teamwork.

"If we have agreed that we have poor facilities then the team culture cannot be that excellent as we have no place to collaborate."

The results of the simulation showed that the space had a major impact on a patient' s health, which led to a discussion of the relationship between the space and many of the variables in the model.

"I believe it is a true picture...you cannot communicate with a patient in a poor space and furthermore the quality of the space has a considerable impact on the quality of the observation and assessment."

### Obstacles, difficulties and progress of the project

Findings from the interviews and the verbatim from the field notes and the video-recording showed that there were some noticeable obstacles and difficulties related to the project, which hampered the collaborative design process, especially at the beginning. Three barriers to the use of SD were revealed, such as an experience of 1) an unclear project presentation and goal-setting 2) an abstract, difficult and time-consuming method and 3) too few experimentation workshops.

#### Unclear project presentation and goal-setting

The first interview showed that the participants considered the aim of the project to be unclear. They wanted more information about the overall aim of the project and a clear picture about the end product (a completed model). They had already started with the design process and were confused about how the modelling project could contribute to the process. Some of them were even anxious that the project would delay the plans for a new stroke unit. Despite this, the participants reported that in general they found the group sessions successful (Table 1). Moreover, they considered the method to be interesting and exciting.

"The method has revealed a new way of thinking which is positive and it has clearly shown the importance of dedicating time to projects like this before discussing detailed design plans at the start."

"We received answers to things we had discussed before."

"To collaborate with other professionals and elucidate new aspects and perspectives."

#### An abstract, difficult and time-consuming method

In the first interview, it was apparent that the participants considered the method to be too abstract and technical to be useful. Additionally, they thought that the method was time-consuming and that the progress of the project was too slow. Several of the participants were also critical about being forced to quantify the relationships in the model. During the initial workshops there were comments from the participants, such as:

"We cannot put figures on our work. It becomes so unrealistic. It is a dynamic world as it is human beings we treat."

This attitude was particularly obvious when the group was asked to quantify the relationships that were qualitative in nature. The participants were required to present in figures the magnitude of some of the relationships of the variables and of their mutual impact on each other. They declined at this workshop, arguing that it was impossible. However, after discussions about the values consensus could be reached.

#### Too few experimentation workshops

According to the interviews, the participants considered the conceptualisation to be too extensive in relation to experimentation. They stated that more time should have been devoted to experimentation with the model since those elements yielded a deeper understanding of SD and the usefulness of modelling. In addition, they stated that an example of a completed model presented to them at the beginning of the project would have facilitated their understanding. These statements were supported by the content of the field notes. During the experimentation it was apparent that the participants acquired a comprehensive understanding of the method.

The participants stated that they developed knowledge about the modelling approach during the course of the project and this was supported by data from the observation and field notes. It was also apparent that the attitudes of the participants were much more positive to using SD at the end of the project than at the beginning due to their growing understanding of the method.

"It was very difficult and abstract at the beginning. I can't understand models. I could not understand what the result should be. But now I understand and realise how useful the model is."

## Discussion

The main findings showed that SD supported the collaborative design process by directing communication in the group towards stroke patients and the stroke care processes. Moreover, the construction of the model forced the group to conceptualise the stroke care process. The findings not only demonstrated that experimentation with the model contributed to an understanding of SD but also that it gave the opportunity to reflect on care. Furthermore, the attitudes to using SD were more positive at the end of the project due to the plausibly participants' growing understanding of the method.

The findings in the present study are important to the future development of the design process, which is in need of greater system thinking. It is necessary for the participants involved to create shared knowledge of the health care environment in interactive discussions [17,18]. Methods that facilitate a dialogue regarding goals for caring, processes and human activities are imperative as it is fundamental that the activity in the future building is defined and discussed in dialogue between the caring professionals and the building planners/architects.

There were some obstacles to the use of SD in the collaborative design process. At the beginning, some of the participants expressed frustration with the project, e.g. that it was difficult and time-consuming. It was a difficult task to introduce a new method that involved technical components in a health care setting. None of the participants were familiar with modelling and simulation. Building a SD model in a group that included a variety of professionals was challenging since the experiences and expectations of building managers and health care professionals were very different. However, the findings showed that the group modelling achieved important goals. The model developed was considered an important and realistic description of the stroke care process. In addition, it was notable that the patient's health and factors that contribute to health in stroke care were constantly in focus in the discussions and the participants' shared viewpoints. This is in agreement with earlier studies, which have shown that group modelling with a "bottom-up" approach, in which the model is developed in collaboration from the very outset, creates commitment to the model [42,43].

Each modelling project benefits from a careful process of acceptance, where the project leader must make sure that everyone in the group agrees on the objectives and limitations of the project [44]. This could have been done more precisely in the present project. The introduction of a "bottom-up approach" along with a demonstration of a fully developed SD model at the first meeting, as was suggested by some participants, might have clarified the purpose of working with concepts and definitions and might be a way to reduce some initial frustration in future design projects.

It is reasonable that the participants found the method time-consuming. A collaborative design approach with the aim of scrutinising the care in order to improve the design takes time, regardless of the method. Professionals involved in building projects are used to focusing strictly on the physical design. In the present project, we initiated discussions about the objectives of patient care and the design solutions involved as important structural variables in improving the quality of the care provided. The aim of the project was not to deliver the final design "solution" but rather to shift the focus beyond direct construction towards system and process issues. This approach could be unfamiliar, which might explain the difficulties expressed by some participants.

The participants stated strongly that they wanted to experiment more with the model. This indicates that they were interested and found the model useful. At the beginning of the project we focused too little on the simulation model, which might have been a hindrance rather than support for the process. At the last two workshops the group showed great understanding of the technical model and how it was constructed, which also contributed to their understanding. Other studies have shown that important learning take place during equation writing and simulation [44,45]. It is thus important that the user of the model has the opportunity to be involved in the whole process. This was also obvious in the present project – the participants actively discussed the results during the simulation workshop and were eager to go beyond the model to analyse the estimation of the relationships. It is therefore essential that experimentation provide a framework for reflection on the simulation results. Each experiment should be designed carefully and time should be allocated for analysis and discussion of the results.

### Methodological considerations

The study is a case study and involved a few individuals. SD interventions and design processes usually involve small groups and the prerequisites are often natural settings since every context creates its own models for its own specific purposes [43]. The present study followed authentic design work and the group size was in accordance with comparable group modelling and design projects [43,46]. All participants were not able to participate in the last interview for work related reasons. The conclusions of the study were supported by several data sources collected throughout the modelling project, which increases the reliability of the findings. However, due to the small size of the groups more research is needed regarding the use of SD in collaborative design work in order to further improve the quality of the design process for health care environments. In addition, using an attitude test pre and post the modelling intervention could have strengthened the findings and should be used in similar future projects.

## Conclusions and further studies

A collaborative design process has been described as an effective approach for discussion of the inherent goal of the design from an end-user perspective (multiprofessional team, patient) and in this process SD is a valuable tool because of the method's potential to focus on patient-centred activities, conceptualising and reflections. SD could be an important decision-making tool since the method reveals mental models, clarifies hypotheses and allows one to perform studies that are impossible to conduct in a real-world setting. The method should be introduced carefully and be focused on experimentation, which contributes to further understanding of the processes/activities that will take place in the space for which the design is intended. By forcing the user of a model to put explicit figures on variables important for their work may increase understanding and the opportunities offered by SD. This will mainly contribute to further analysis of the modeled system, which is the central purpose of using SD.

An important question in the future is whether the design process and the subsequent quality of the new environment will be improved as a result of a modelling process. Enhanced understanding of the system strengthens the likelihood of successful improvements in an organisation. However, the best evaluation of the benefits of the model is the quality of the decisions that result from the modelling process [47]. Consequently, a combined study design with action research and SD should be realised to evaluate the contribution of SD in a broader sense. Important issues would be how to integrate various modelling approaches into the design process as well as other quality improvement technologies, such as Deming' s [48] Plan-Do-Study-Act (PDSA) cycle.

## List of abbreviations

SD – system dynamics

CLD – causal loop diagram

SFD – stock and flow diagram

PDSA – Plan-Do-Study-Act

ICF – International classification of functioning, disability and health

## Appendix 1

In order to design and test different policies, the group gradually developed a stroke care model. The description of the model is a dynamic hypothesis of the stroke care process. The patient's health status and the care plan are stocks in the model, which can be filled or drained according to the quality of the other variables. A patient's health status is a function of the quality of the care interventions the team managed to give to the patient, which are in turn influenced by the quality of the observation and assessment performed by the professionals [49]. The assessments of a stroke patient's health should include intellectual and cognitive capacity, emotional disturbance and motivation, and should cover the degree of motor weakness and sensory and visual loss. Moreover, the patient's nutrition, skin, and activity status must be assessed and evaluated on a daily basis [50]. According to the model, an increase in the quality of the observation and assessment will lead to an increase in the quality of delivered interventions, which will in turn enhance the patient's health [49]. Improved health as a whole will increase the potential to further improve the quality of observation and assessment.

The model suggests that communication is an important factor in stroke care, both for the potential to make an accurate assessment of the patient's needs and health problems and also to bring about patient involvement and influence on her/his own care [51,52]. If the quality of communication between the care professionals and the patient increases the model hypothesises that patient involvement will also increase. This will in turn have an influence on the patient's recovery time and consequently on the patient's health.

A care plan is a written document in the patient's records for those team members who meet the patient throughout the care period in order to assist in monitoring their contact with the patient [53,54]. A care plan should illustrate decision-making in patient care, which is an essential process in modern health care. This process involves judgments (diagnosis) of the patient's health and decisions about care interventions (management of care) [55].

There is a mutual influence between observation and assessment and the care plan. More accurate observation and assessment will improve the quality of the care plan and an adequately written care plan increases the quality of the observation and assessment [55].

A written care plan is also hypothesised to facilitate teamwork. The increased quality of the care plan influences the teamwork in the sense that the discussions in the team will be more focused on the individual patient's health problem. It is also more likely that the various professionals' contributions will be more explicit and discussed more. High-quality teamwork increases the quality of observation and assessment of the patient's health since the collaborative analysis of the patient's health problem may contribute to a broader understanding of the problem and thus contributes to closer observation of nuances in the patient's health.

The care culture influences the quality of communication with the patient and relatives since a strong care culture support factors that facilitate patient-centred care [56]. Professional knowledge has been suggested as a factor that has been shown to contribute to the significant differences between stroke units and general medical wards. The model indicates that the quality of the physical space directly influences the recovery time and thus the patient's health status [57].

## Competing interests

The author(s) declare that they have no competing interests.

## Authors' contributions

Marie Elf has been the project leader and has primary responsibility for the formulation of the study. She has conducted the studies/data collection, as well as data analysis. She has presented, interpreted and discussed the results in the manuscript. Mariya Putilova, supported Marie in the modelling process and interpretation of the results. Kerstin Öhrn and Lena von Koch were co-authors and supported Marie in the analysis, interpretation of results and scientific writing.

## Pre-publication history

The pre-publication history for this paper can be accessed here:


